# Adult Women First Exposed to Early Adversity After 8 Years Old Show Attentional Bias to Threat

**DOI:** 10.3389/fnbeh.2021.628099

**Published:** 2021-05-04

**Authors:** Catherine Raymond, Marie-France Marin, Victoria Wolosianski, Audrey-Ann Journault, Charlotte Longpré, Sonia J. Lupien

**Affiliations:** ^1^Center for Studies on Human Stress, Institut Universitaire en Santé Mentale de Montréal, Research Center, CIUSSS Est-de-l’Île-de-Montréal, Montréal, QC, Canada; ^2^Department of Neurosciences, Université de Montréal, Montréal, QC, Canada; ^3^Department of Psychology, Université du Québec à Montréal, Montréal, QC, Canada; ^4^Department of Psychology, Université de Montréal, Montréal, QC, Canada; ^5^Department of Psychiatry and Addictology, Université de Montréal, Montréal, QC, Canada

**Keywords:** early adversity, minimal age at exposure, attentional biases, emotion regulation, declarative memory

## Abstract

Exposure to early adversity (EA) is associated with long-lasting dysregulations in cognitive processes sustained by brain regions that are sensitive to stress hormones: the hippocampus, the amygdala, and the prefrontal cortex. The Life Cycle Model of Stress highlights the importance of considering the timing at which EA began, as these brain regions follow distinct developmental trajectories. We aimed to test this hypothesis by assessing whether adults exposed to EA exhibit different cognitive patterns as a function of the age at which they were first exposed to EA. Eighty-five healthy men and women aged 21–40 years old (y/o) exposed to EA, as assessed by the Adverse Childhood Experience Questionnaire, were grouped based on the age of first exposure to EA: 0–2 y/o (“Infancy”: hippocampal development), 3–7 y/o (“Early childhood”: amygdala development) and after the age of 8 (“Childhood/Adolescence”: frontoamygdala connectivity development). Declarative memory, attentional bias to threat and emotion regulation were measured. Results revealed increased attentional bias to threat in women first exposed to EA after 8 years. This result is in line with the Life Cycle Model of Stress and highlights the importance of considering the age at exposure to EA when investigating the effects of EA on cognitive processes.

## Introduction

Exposure to early adversity (EA) is associated with an increased incidence of various psychopathologies associated with dysregulated emotional and cognitive processes such as depression and anxiety (for a review, see Gershon et al., 2013), which are twice as prevalent in women compared to men (Bangasser and Valentino, 2014). By its very nature, EA leads to prolonged stress, increasing the secretion of stress hormones at an early age (cortisol in humans; Carlson and Earls, 1997; Fisher et al., 2000; Kertes et al., 2008; Bruce et al., 2009; Bernard et al., 2015).

Being liposoluble, cortisol easily crosses the blood-brain-barrier and binds to multiple brain regions, notably those that support cognitive processes that are crucial in stress regulation: the hippocampus (Herman et al., 2012), the amygdala (van Stegeren et al., 2007), and the prefrontal cortex (Diorio et al., 1993). The hippocampus is important for explicit (i.e., declarative) memory formation and in the consolidation (Morris, 2007) and contextualization (Wiltgen et al., 2006) of information to be encoded. The amygdala is responsible for the detection of negative emotions and for threat detection (Bishop, 2008), and the prefrontal cortex plays a key role in emotion regulation processes through its functional inhibitory connection to the amygdala (frontoamygdala connectivity; Wager et al., 2008). These brain regions also play a critical role in the regulation of the physiological stress system: the hippocampus (Herman et al., 2012) and the prefrontal cortex (Diorio et al., 1993) both inhibit it, whereas the amygdala activates it (Herman et al., 2003).

A wealth of studies have assessed cognitive functions sustained by these regions in children and adults exposed to EA (for a review, see Lupien et al., 2009). Results show impaired declarative memory in trauma-exposed children (Ayoub et al., 2009; Bos et al., 2009; Carrión et al., 2010), and increased threat detection in children exposed to trauma relative to non-exposed children (Pollak et al., 2000; Fries and Pollak, 2004; Cicchetti and Curtis, 2005; Parker and Nelson, 2005). A study reported that this effect may be amplified in girls as opposed to boys (Soe et al., 2018). For prefrontal cortex and frontoamygdala connectivity functions, studies reported decreased abilities of emotion regulation in children exposed to trauma (Maughan and Cicchetti, 2002; Marusak et al., 2015; McLaughlin et al., 2015). All these effects seem to be long lasting, with studies reporting decreased declarative memory (Majer et al., 2010), increased threat detection (Nicol et al., 2014; Russo et al., 2015), and decreased emotion regulation abilities (Abravanel and Sinha, 2015) in clinical samples of adults reporting EA during childhood. However, so far, this pattern of altered cognition in adulthood has mostly been reported in subjects suffering from a psychopathology (such as anxiety or depression; Clark et al., 2010), making it difficult to determine whether the impaired cognitive processes result from EA or from the pathology.

Interestingly, these three brain regions that are sensitive to stress hormones follow different developmental trajectories. While the volume of the hippocampus expands from birth until the age of 2, the amygdala develops from the first year of life until the age of 20, and the prefrontal cortex as well as the frontoamygdala connectivity mainly develop between the ages of 8 and 29 (Giedd et al., 1996; Lupien et al., 2009). The age of 8 years old therefore represents an important milestone in brain development, as the establishment of the frontoamygdala connectivity allows for the independent inhibition of the amygdala by the prefrontal cortex during threat perception (Lupien et al., 2009). Recent studies have shown that before the development of the frontoamygdala connectivity (i.e., before 8 years old), the parent plays a critical role in regulating the child’s stress response during threat perception, although this “buffering effect” is dampened when being reared in early adverse conditions (Gunnar et al., 2015).

The differential development of the brain regions sensitive to stress hormones has led to the “Life Cycle Model of Stress”, which suggests that there may be early windows of vulnerability during which specific regions of the developing brain are most sensitive to stress hormones produced in response to environmental influences (Lupien et al., 2009). Exposure to stress and/or adversity during these key vulnerable periods could modulate the development of those brain regions for the duration of the adversity. The Life Cycle Model of Stress implies that the age at which an individual is exposed to EA for the first time (namely, the “minimal age at exposure”) could lead to different physiological and/or cognitive outcomes depending on the brain regions that are developing at the time of exposure.

Given the importance of the hippocampus, the amygdala and the prefrontal cortex on regulating the activity of the physiological stress system (Lupien et al., 2009), in a first study, we tested the Life Cycle Model of Stress by measuring the effects of minimal age at exposure on the activity of diurnal and reactive cortisol levels (Raymond et al., 2021). We also compared this model (i.e., minimal age at exposure) to the classical “Accumulation model” of EA, which stipulates that the number of EA predicts patterns of cortisol dysregulations in adulthood. Results showed that although the number of EA was not associated with patterns of basal or reactive cortisol secretion, adults first exposed to EA between the ages of 3 and 7–an important time window for amygdala development–showed greater cortisol awakening response and lower cortisol reactivity relative to those first exposed to EA before 3 or after 7 (Raymond et al., 2021).

The minimal age at exposure could also be used to predict the nature of the cognitive patterns that will result from EA. For example, first exposure to adversity during the first year of life (hippocampal development) could result in later (adult) declarative memory impairment, whereas first exposure to adversity at the age of 7 years old (amygdala development) would result in increased threat perception. On the other hand, exposition to EA at 10 years of age could impact prefrontal cortex and lead to decreased frontoamygdala connectivity development that would translate in difficulties in emotion regulation in adulthood. Although the effects of EA on cognitive functions of children and adults have been assessed in previous experiments, no study to date has tested the effects of minimal age at exposure to EA on cognitive functions sustained by the hippocampus, the amygdala, and the frontoamygdala connectivity in adulthood.

The purpose of the current study was to test the Life Cycle Model of Stress by measuring the effects of minimal age at exposure on cognitive processes of healthy adults as a function of whether individuals were first exposed to EA during the development of the hippocampus, the amygdala and the frontoamygdala connectivity. The Life Cycle Model of Stress predicted that (1) individuals first exposed to EA during the development of the hippocampus would present decreased declarative memory performance in adulthood; (2) individuals first exposed to EA during the development of the amygdala would present increased threat detection in adulthood compared to the other groups, and (3) individuals who were first exposed to EA during the development of the frontoamygdala connectivity would present decreased emotion regulation abilities in adulthood as opposed to the other groups. Furthermore, given the sex-discrepancy in stress-related psychopathologies in adulthood, we expected that these effects would be greater in women as opposed to men.

## Materials and Methods

### Participants

Fifty-three naturally cycling women and 32 men aged 21–40 (*M* = 28.28, ±5.56) participated in a two-session protocol, occurring 14 days apart. Participants were recruited from the greater Montreal region through advertisements asking for healthy adults exposed to diverse forms of EA. Internet advertisements and posters on University campus were used. Interested participants contacted a study staff member and were screened over the phone to make sure that they did not suffer from any physiological (neurological, cardiovascular disease, and general health problems) or psychological (ex. diagnosed depression, schizophrenia, and personality or anxiety disorders) conditions that could influence the results. Participants did not take medication and oral contraceptive use was also an exclusion criterion for women. Participants were screened for EA over the phone (see section “Assessment of early adversity: Adverse Childhood Experience Questionnaire”). Eligible participants were then scheduled for two visits at the laboratory, at a 2-weeks interval period, between 1:30 p.m. and 4:30 p.m to control for the circadian cycle of cortisol and for synchrony effects on cognition (May and Hasher, 1998).

Moreover, given that we also took measurements of physiological stress (results published in Raymond et al., 2021), all women performed the first testing session during the follicular phase of their menstrual cycle (based on women self-reports on their last menstruation and their menstrual cycle length) given the known interaction between the hypothalamic-pituitary-adrenal axis and the hypothalamic-pituitary-gonadal axis. This procedure was implemented so that women would be in the luteal phase of their menstrual cycle 2 weeks later when they back to the laboratory for a follow-up session where they underwent the *Trier Social Stress Test* in order to test their cortisol reactivity, and given the well-known effects of sex hormones on salivary free cortisol levels (Kirschbaum et al., 1999).

### Questionnaires

#### Assessment of EA: Adverse Childhood Experience Questionnaire

The international version of the Adverse Childhood Experiences Questionnaire (ACE-Q; World Health Organization, 2014) was administered to all eligible participants to assess exposure to EA. The ACE-IQ, which was administered during phone screening, is a validated 13-item questionnaire assessing physical, sexual, and emotional abuse, neglect, environmental disaster, household dysfunctions such as witnessing violence, parental separation, death or mental illness, substance abuse, bullying, as well as collective and community violence. The participant must answer “yes” or “no” to each item. To assess the minimal age at exposure to EA, we added one question to every item of the ACE-IQ. Therefore, participants answering that they were exposed to a subtype of adversity were asked to specify at what age the specific EA type began. In order to calculate the time elapsed since last exposure, participants were also asked at what age it last happened (which was calculated as the subtraction of the age at last exposure from the age at the time of testing).

Given the fact that exposition to EA has been shown to lead to various stress-related psychopathologies associated with decreased emotion regulation abilities in adulthood, we compared the three groups on depressive symptoms, trait anxiety, as well as explicit (i.e., trait) emotion regulation strategies using the following questionnaires.

#### Beck Depression Inventory

Depressive symptoms were assessed with the Beck Depression Inventory-II (BDI-II; Beck et al., 1988), which is a 21-item self-reported questionnaire assessing depressive symptoms that occurred in the past 2 weeks. The BDI assesses depressive symptoms with statements ranging from 0 to 3 in terms of intensity. A meta-analysis showed that this instrument has a test–retest reliability ranging from *r* = 0.60–0.83, and a Cronbach’s alpha of 0.81 for non-psychiatric participants (Beck et al., 1988). In this study, we used the sum score of the BDI-II, with higher scores indicating more severe symptoms.

#### State-Trait Anxiety Inventory for Adults

Trait anxiety was assessed with the State-Trait Anxiety Inventory for adults (STAI-Y; Spielberger, 1983). This questionnaire consists of 40 items and is divided into two subscales: Trait and State anxiety. The Trait subscale measures anxiety as a personality trait. As a result, these questions must be answered in a general sense and not with regards to a particular situation. This subscale consists of 20 questions that are answered on a scale ranging from 1 to 4 (1 meaning « hardly ever » and 4 being « almost always »), where a high score indicates a high level of anxiety in general. In this study, only the sum score of the Trait subscale was used. The test–retest reliability for the Trait subscale is *r* = 0.86 and the internal consistency is 0.90 (Spielberger, 1983).

#### Affective Style Questionnaire

In order to measure dispositional emotion regulation, participants filled out the Affective Style Questionnaire (Hofmann and Kashdan, 2010). The ASQ is a 20-item instrument that measures individual differences in emotion regulation abilities. Each item is measured on a 5-point Likert scale. The questionnaire is composed of three subscales: “Concealing” (which refers to habitual attempts to conceal or suppress affect), “Adjusting” (a general ability to manage, adjust, and work with emotions as needed), and “Tolerating” (an accepting and tolerant attitude toward emotion). The scale is reliable (*r* = 0.062) and has good internal consistency (Concealing α = 0.84, Adjusting α = 0.82, and Tolerating α = 0.68).

### Cognitive Assessment

To answer our primary research questions, we selected cognitive tasks sustained by the three brain regions involved in stress regulation (hippocampus, amygdala, and prefrontal cortex) and that are therefore likely to be influenced by chronic stress following exposure to EA.

Each task was programed using E-Prime (Version 3.0; Psychology Software Tools, 2016) and was conducted on a Dell Inspiron 6000, 400 MHz laptop computer with a 15-inch monitor. Observations were made in a seated position, at a distance of 60 cm from the screen.

#### Declarative Memory

A word list of 20 neutral words controlled for word length, frequency of use and grammatical category was created by our team. This type of words list has been commonly used to assess declarative memory performance in other studies (Eichenbaum, 2004; Bird and Burgess, 2008; Canamar and London, 2012; Jeneson and Squire, 2012). Each trial began with a fixation cross presented for 500 ms, followed by the stimulus (i.e., word) for 2,000 ms. Words were presented one by one, printed in black (*Arial*, font size 24) and presented twice randomly. Three free recalls were conducted: (1) immediately after word presentation; (2) 20 min later, and; (3) 2 weeks later during the second testing session. Between the immediate and the +20 min recall, participants were asked to read magazines, waiting for the next task. At each recall phase, participants were given 5 min to remember as many words as possible and write them down. This type of task has been shown to rely on hippocampal activity.

#### Attentional Bias Toward Threat

In order to assess attentional bias toward threat (vs. neutral ones), we used a modified version of the *Posner spatial orienting paradigm* (Posner, 1980) that we previously used in another study assessing attentional biases (for task overview, see Pilgrim et al., 2010). The task began with a black cross on a white background, which remained on the screen. Afterward, a word (Arial black font, point size of 24) was presented for 200 ms on either the right or left hand side of the screen. This word was either positive (i.e., calm), threatening (i.e., judged), or neutral (i.e., measure) and served as a stimulus cue to the location of a subsequent target. A black target asterisk appeared immediately after the word cue disappeared and remained on the screen for 1,000 ms. Subjects were told to respond as quickly and accurately as possible regarding the spatial location of the target. They pressed one key if it appeared on the right (M) side of the black cross and another if it was shown on the left (Z).

Given that the main purpose of this task was to verify whether groups differed in terms of their attentional biases toward threatening cues as opposed to neutral ones, we only analyzed the valid trials for threatening and neutral cues. Hence, when negative words related to threat were previously presented, a more rapid response (as opposed to neutral ones) on the target indicated an attentional bias toward threatening information. As such, the reaction time for negative words was subtracted from the reaction time to neutral words to create a ratio (Pilgrim et al., 2010). Hence, a negative ratio indicates an attentional bias toward threat.

There was a total of 540 trials with 360 valid trials (66%), i.e., when the cue and the target appeared on the same side of the display, assessing attentional engagement. There were 180 invalid trials (33%), i.e., when the cue and the target appeared on opposite sides of the display, examining attentional disengagement. An additional 48 animal word cue trials were embedded within regular trials and participants were instructed to refrain from responding. These animal word cue trials were included: (1) to reduce the generation of automaticity and (2) to increase the degree of attention paid to the semantic nature of words (these were not analyzed in the current study). Another 15 separate practice trials (Ekman, 1976) incorporating different word cues than the experimental trials were presented at the start of the task. This type of task measuring attentional biases toward threat (vs. neutral ones) trough reaction time measurements has been shown to rely on the activity of the amygdala (Goldstein et al., 1996; Pasley et al., 2004; Vuilleumier and Pourtois, 2007; Tottenham and Sheridan, 2010; Méndez-Bértolo et al., 2016; Mier et al., 2017).

#### Emotion Regulation

**FIGURE 1:**
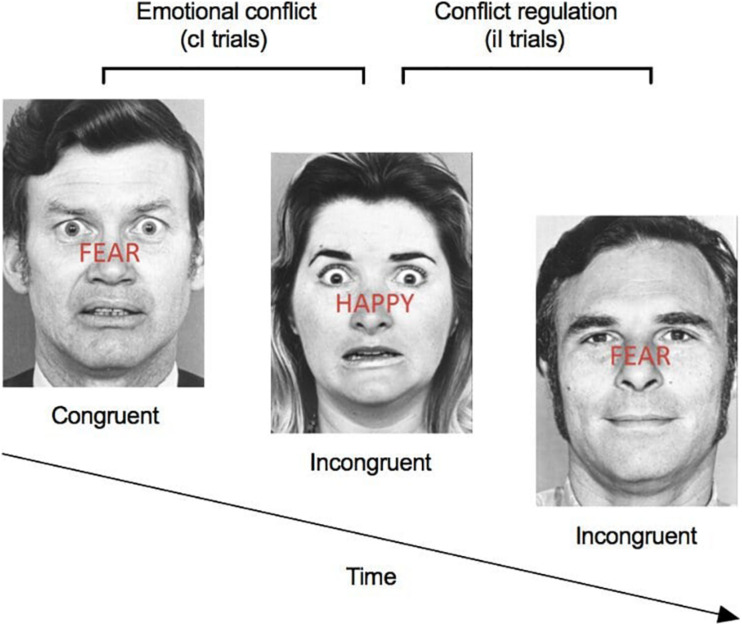
Emotional conflict task. Participants were instructed to identify as quickly and accurately as possible the underlying facial emotion (happy or fearful), while inhibiting the reading of the overlying word (happy or fear). Trials varied in congruency such that some trials were “congruent” (i.e., non-conflict, expression of the actor matched the written expression) and others were “incongruent” (i.e., conflict, expression of the actor did not match the written expression). One-hundred sixty (160) trials were presented: half were congruent and half were incongruent. Conflict regulation was measured by contrasting reaction time for “congruent followed by incongruent” trials (cI trials) to “incongruent followed by incongruent” trial (iI trials).

The automatic regulation of emotion processing was assessed with the *Emotional conflict task* (Etkin et al., 2015). The task consisted of 160 presentations of happy or fearful facial expression photographs drawn from the set of Ekman and Friesen (Ekman, W.V. Friesen, Pictures of Facial Affect, Consulting Psychologists, Palo Alto, CA; 1976). Photographs were overlaid with “HAPPY” or “FEAR” written in red letters (see Figure 1 for task overview). Subjects were instructed to identify as quickly and accurately as possible the underlying facial emotion of visual stimuli (happy or fearful), while inhibiting the reading of the overlying word (happy or fear). Trials varied in congruency in such a way that some trials were “congruent” (i.e., non-conflict, expression of actor matched the word) and others were “incongruent” (i.e., conflict, expression of actor did not match the word). One hundred and sixty (160) trials were presented: half (80 trials) were congruent, and the other half (80 trials) were incongruent. As previous studies have demonstrated, incongruent trials induce an emotional conflict, which is associated with slower reaction times, increased activation of the prefrontal cortex, and decreased activation of the amygdala (Etkin et al., 2015). Stimuli were presented for 1,000 ms, with a varying interstimulus interval of 2,000–4,000 ms (mean = 3,000 ms), in a pseudorandom order and were counterbalanced across trial types for expression, overlying word and sex of figures. Response times were included in the analyses for all correct trials with a reaction time between 200 and 1,200 ms (see Figure 1 for task overview). Conflict regulation was measured by contrasting reaction time for “congruent followed by incongruent” trials (cI trials) to “incongruent followed by incongruent” trial (iI trials). This kind of task has been shown to rely on the top-down inhibition of the amygdala by the prefrontal cortex, both critical structures involved in the emotion regulation system (Etkin et al., 2015) and in stress response (Lupien et al., 2009).

### General Protocol

The ethics committee of the *Research Center of the Institut universitaire en santé mentale de Montréal* approved this study and all participants provided written informed consent to take part in the study. Upon their arrival at the laboratory for their first visit, and after giving their written consent, participants were first asked to complete the cognitive tasks (declarative memory, modified version of the *Posner paradigm*, and the *Emotional conflict task*). In order to control for a potential fatigue effect, the order of the tests was randomly counterbalanced between participants. Following the first session, participants were asked to complete the questionnaires (BDI-II, STAI-T, ASQ, and others that were not analyzed in the current study) at home via the Studies Web Automation Tool (SWAT), which is a secured web platform developed by the Center for Studies on Human Stress (Montreal, QC, Canada). Since we wanted to ensure confidentiality, participants were given individualized secure codes to log in and access the online questionnaires.

After 2 weeks, participants were asked to come back to the laboratory at the same time as their first visit that had occurred 14 days earlier. Upon their arrival at the lab, they were asked to remember as many words as possible from the first session (third free recall of the declarative task; see section State-Trait Anxiety Inventory for Adults). Participants thereafter completed additional tasks during this session, though they will not be discussed within the context of the current paper.

### Initial Treatment of the Data

*Group formation: Minimal age at exposure to EA*: Based on the information collected during the phone interview, we categorized the participants in three different groups based on the age at which the first EA occurred: between 0 and 2 years old (during hippocampal development), thereafter named “Infancy”; between 3 and 7 years old (during amygdala development), thereafter named “Early childhood” or after 8 years old (frontoamygdala connectivity development) thereafter named “Childhood/Adolescence”. In a first study, we have demonstrated that this model predicted both basal and reactive cortisol levels, and that this model was a better statistical fit as opposed to the classical “Accumulation” model of EA which stipulates that the number of EA predicts patterns of cortisol dysregulations in adulthood (see Raymond et al., 2021; for statistical details and Goodness of fit indices).

### Statistical Analyses

Data were examined for potential outliers via studentized residuals, with residuals ≥±3.29 considered outliers. Two participants exhibited extreme score on the Posner task in the 0–2 years old group (one above and one below average). One participant exhibited extreme (above average) scores on the BDI in the 8+ years old group and two participants on the STAI-T in the 3–7 years old group (one above and one below average). In line with ethical considerations, participants who had a score of 14 or more on the BDI (criteria for mild depression; Beck et al., 1988) were contacted by the main investigator and provided with psychological resources. Analyses were run twice: once including the Winzorized values, and once excluding them. Since no difference was found between the two sets of analyses, Winzorized data were included in the final analyses. The distribution of our variables was also assessed for skewness and kurtosis prior to conducting the statistical analyses. Using indices for acceptable limits of ±2, data were found to be normally distributed (George and Mallery, 2010).

First, we conducted an ANOVA to assess whether groups differed in terms of their scores on the BDI-II, STAI-T and the three subscales of the ASQ. For cognitive processes, we first assessed whether groups (3) differed on overall task accuracy and reaction time (modified version of the *Posner paradigm* and *Emotional conflict task*). Thereafter, we conducted an ANOVA for attentional biases (reaction times in the modified version of the *Posner paradigm*) and on the conflict regulation ratio (reaction time for iI–cI) with groups (3: minimal age at exposure 0–2 years old; 3–7 years old – +8 years old) and sex (2: women and men) as the between subject factors. We also verified whether groups differed in declarative memory using a repeated measures ANOVA with time (3 recalls: immediate – +20 min – +2 weeks) as the within subject factor and groups (3: minimal age at exposure 0–2 years old; 3–7 years old – +8 years old) and sex (2: women and men) as the between subject factors. Significant interactions were decomposed and Bonferroni corrections were applied when multiple comparisons were conducted during *post hoc* analyses.

## Results

### Preliminary Analyses

In our sample, the mean score on the ACE-IQ was 4.6 (min = 1; max = 11; ±2.25). Analyses confirmed that each group differed from each other in minimal age of exposure [F(2,84) = 223.450; *p* < 0.001] (all *p*s < 0.001). Analyses also revealed group differences in accumulation of EA (ACE-Q summed score) [F(2,84) = 5.119; *p* = 0.008], with the “Childhood/Adolescence” group presenting lower accumulation of EA as opposed to the “0–2 years old” group (*p* = 0.007). Groups did not differ on the time elapsed since last exposure to EA [F(2,84) = 0.698; *p* = 0.501], nor in age [F(2,84) = 0.272; *p* = 0.763] (see Table 1). Given group differences on the ACE-Q, and to ascertain that obtained group differences as a function of minimal age at exposure did not pertain to this potential confounding factor, we re-conducted every significant analyses while including the ACE-Q summed score as a covariate.

**TABLE 1:** Demographic information and health behaviors as a function of minimal age at exposure.

	**Infancy**	**Early childhood**	**Childhood/Adolescence**	***p***
*N* (women)	30 (23)	25 (15)	30 (15)	
Age	27.73 (5.20)	28.32 (6.11)	28.80 (5.58)	0.575
Minimal age at exposure to EA	0.27 (0.64)*	4.36 (0.95)*	10.10 (2.84)*	<0.001
ACE-Q sum score	5.36 (2.20)	4.84 (2.49)	3.63 (1.73)*	0.008
**Health behaviors**				
Alcohol intake (# of drinks/week)	3.25 (3.91)	2.40 (3.72)	2.79 (2.76)	0.773
BMI	24.94 (3.32)	23.03 (2.71)	24.25 (3.73)	0.250

Each panel depicts the number of participants for each group (with the number of women), as well as the mean (and corresponding standard deviation) for the following variables: age (in years), minimal age at exposure to EA (in years), sum score to the ACE-Q. Asterisk (*) indicates significant between-group differences. ACE-Q, Adverse Childhood Experience Questionnaire; EA, early adversity; MAE, minimal age at exposure; SD, standard deviation.

#### Depressive Symptoms

The ANOVA revealed no main effect of group [F(2,84) = 1.640; *p* = 0.201], nor a group × sex interaction [F(2,84) = 0.600; *p* = 0.552] (see Table 2 for further details).

**TABLE 2:** Socio-emotional characteristics as a function of minimal age at exposure and sex.

	**Infancy**		**Early Childhood**		**Childhood/Adolescence**		***p***
	**Men**	**Women**	**Men**	**Women**	**Men**	**Women**	
Beck depression inventory-II	11.57 (2.84)	10.09 (1.64)	6.33 (2.50)	9.64 (2.00)	6.29 (2.01)	7.53 (1.94)	0.580
State trait anxiety inventory - trait	47.57 (1.51)	49.76 (0.87)	50.56 (1.33)	50.29 (1.07)	50.93 (1.09)	49.93 (1.03)	0.369
**Affective style questionnaire**							
Concealing	28.00 (2.50)	23.67 (1.34)	27.67 (2.21)	21.36 (1.77)	23.14 (2.98)	24.93 (1.70)	0.079
Adjusting	17.43 (2.08)*	21.53 (1.20)	23.56 (1.83)	19.79 (1.47)	23.79 (1.90)	21.27 (1.42)	0.039*
Tolerating	16.71 (1.14)	16.90 (0.66)	17.00 (1.01)	15.64 (0.81)	17.64 (0.98)	16.27 (0.78)	0.612

No group differences were found on the Beck Depression Inventory, the Trait subscale of the State Trait Anxiety Inventory nor on the three subscales of the Affective Style Questionnaire. A group by sex interaction was found on the Adjusting subscale of the Affective Style Questionnaire. *p < 0.05. Mean (SD).

#### Trait Anxiety

The ANOVA revealed no main effect of group [F(2,84) = 1.224; *p* = 0.300], nor a group × sex interaction [F(2,84) = 0.999; *p* = 0.373] (see Table 2 for further details).

#### Trait Emotion Regulation

**FIGURE 2:**
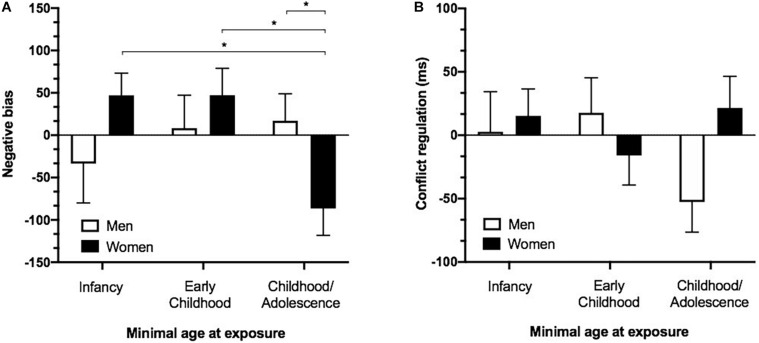
Attentional biases and emotion regulation as a function of minimal age at exposure and sex. **(A)** Increased attentional biases toward threatening information in women in the “Childhood/Adolescence” group as compared to men in the “Childhood/Adolescence” group, women in the “Early childhood” and women in the “Infancy” group. A negative value indicates a faster reaction time to negative words relative to neutral words and therefore suggests an attentional bias toward threat. **(B)** Emotion conflict regulatory ability in individuals who were first exposed to EA during “Infancy” (0–2 years old), “Early childhood” (3–7 years old) or “Childhood/Adolescence” (after 8 years old). Negative values indicate faster response for iI relative to cI trials (iI-cI) and suggest appropriate emotion regulation. *p < 0.05. Error bars represent SEM. MEA, minimal age at exposure; y/o, years old.

For the “concealing” subscale of the ASQ, the MANOVA revealed no main effect of group [F(2,84) = 0.432; *p* = 0.651], nor a group × sex interaction [F(2,84) = 1.949; *p* = 0.150]. For the “adjusting” subscale of the ASQ, the MANOVA revealed no main effect of group [F(2,84) = 1.871; *p* = 0.161], but a group × sex interaction [F(2,84) = 3.461; *p* = 0.036]. *Post hoc* analyses revealed group differences in men [F(2,32) = 3.661; *p* = 0.039], with those in the 0–2 years old group presenting decreased scores on the “adjusting” subscale as opposed to those in the 3–7 (*p* = 0.032) and 8+ (*p* = 0.016) years old groups, which did not differ from one another (*p* = 0.921; see Figure 2). No group differences were found in women [F(2,52) = 0.441; *p* = 0.646]. For the “tolerating” subscale of the ASQ, the MANOVA revealed no main effect of group [F(2,84) = 0.457; *p* = 0.635], nor a group × sex interaction [F(2,84) = 0.592; *p* = 0.556] (see Table 1 for further details).

### Cognitive Processes

#### Declarative Memory

**FIGURE 3:**
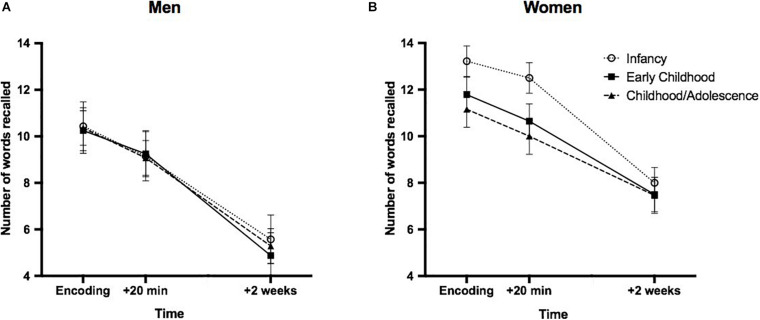
Performance on the declarative memory task as a function of minimal age at exposure and sex. Declarative memory in men **(A)** and women **(B)** first exposed to EA during “Infancy” (0–2 years old), “Early childhood” (3–7 years old) or “Childhood/Adolescence” (after 8 years old). A main effect of sex was found, with women presenting an overall increased word recall as opposed to men. Error bars represent SEM.

The ANOVA performed on declarative memory performance revealed a significant main effect of time [F(2,136) = 234.557; *p* < 0.0001], with each timepoints differing from one another (encoding: *M* = 11.38, SD = 2.85; +20 min: *M* = 10.33, SD = 2.95; 2 weeks post encoding: *M* = 6.73, = SD; all ps < 0.0001). A main effect of sex was found [F(2,68) = 0.535; *p* = 0.002], with women presenting increased recall at all timepoints as opposed to men (Figures 3A,B). No main effect of group [F(2,68) = 0.803; *p* = 0.452], no time × group interaction [F(4,136) = 0.738; *p* = 0.568], nor a time × group × sex interaction [F(4,136) = 1.308; *p* = 0.275] was found.

#### Attentional Bias Toward Threat

The ANOVA revealed no main effect of group [F(2,84) = 1.684; *p* = 0.192], but a significant group × sex interaction [F(2,84) = 3.785; *p* = 0.027]. *Post hoc* analyses revealed group differences in women [F(2,52) = 4.349; *p* = 0.018], with women in the Childhood/Adolescence group presenting greater attentional bias toward threat as opposed to the Infancy (*p* = 0.01) and Early childhood (*p* = 0.017) groups, which did not differ from each other (*p* = 0.998). No group differences were found in men [F(2,32) = 0.924; *p* = 0.408] (Figure 2A). We re-conducted the analysis while including the ACE-Q summed score as a covariate, which did not modify the obtained group × sex interaction [F(2,83) = 3.734; *p* = 0.028]. As shown in Figure 2A, a negative value indicates a faster reaction time to negative relative to neutral words, indicating an attentional bias toward threat.

#### Emotion Regulation

As expected, responses to congruent trials were significantly faster as opposed to incongruent trials [*t*(93) = 58.543, *p* < 0.0001]. However, the ANOVA revealed no main effect of group [F(2,84) = 0.833; *p* = 0.439] nor a group × sex interaction [F(2,84) = 2.901; *p* = 0.061] (Figure 2B).

## Discussion

The purpose of this study was to test whether the cognitive processes sustained by the brain regions involved in the stress response are modulated by the age at first exposure to EA. We found that although groups did not differ on declarative memory performance and emotion regulation, women who were first exposed to EA after the age of 8 (“Childhood/Adolescence”: during the development of frontoamygdala connectivity) presented increased attentional bias toward threat as opposed to those who were first exposed between 0 and 2 years old (“Infancy”: during the development of the hippocampus) or between 3 and 7 years old (“Early childhood”: during the early development of the amygdala).

The absence of a significant group difference in declarative memory performance could be taken as suggesting that declarative memory is not associated to the age at first exposure to EA. Yet, a few studies found altered verbal memory in traumatized children (Bos et al., 2009) and in adult women exposed to EA and suffering from post-traumatic stress disorder (Bremner et al., 2003, 2004). However, these results were obtained in clinical populations, raising the question as to whether the memory deficits observed were due to exposition to EA or to the underlying psychopathology. The absence of a control group in our study prevents us from answering this important question. Be this as it may, the absence of a group difference in declarative memory performance could also be explained by a number of factors. First, they could be due to the task that we used. It is possible that the word list was not difficult enough given that we recruited resilient/healthy participants (none of them were suffering from a psychopathology) and that 62% of them came from University settings, which promotes the retention of verbal material (Guerra-Carrillo et al., 2017). Another possibility is that our negative finding on the declarative memory task is due to a practice effect, especially in women as they presented greater memory performance when compared to men. It would be interesting to replicate these results with a different task (such as spatial memory), that is still associated with declarative memory.

Interestingly, we found that adult women who were first exposed to EA after the age of 8 presented increased attentional bias toward threat as opposed to the other groups. In line with the Life Cycle Model of Stress (Lupien et al., 2009) one possible interpretation for this finding may be that first exposure to EA during the development of the prefrontal cortex (while the frontoamygdala connectivity is taking place), could result in a diminished capacity of the frontal cortex to inhibit the amygdala during threat perception. Although attentional biases to threat are typically thought to rely on the activity of the amygdala alone, neuroimaging studies have shown that the prefrontal cortex plays a critical role in down-regulating the activity of the amygdala during the processing of threatening stimuli (Browning et al., 2010; Angelidis et al., 2018), allowing disengaging the attention away from threat (Peers et al., 2013). In individuals who first undergone EA *after* 8 years of age, a reduced frontoamygdala functional connectivity may limit the inhibition of the amygdala and result in increased attentional biases toward threat. In support of this idea, recent neuroimaging studies found reduced frontoamygdala connectivity in adolescents (Cisler, 2017; Park et al., 2018) and adults exposed to EA (Javanbakht et al., 2015), although the age at first exposure to EA was not taken into account in these studies. Soe et al. (2018) also found that a reduced frontoamygdala connectivity was more prominent in girls as opposed to boys exposed to EA, which could explain the obtained sex differences in our sample.

The increased attentional bias in women first exposed to EA after the age of 8 is also in line with the Stress Acceleration Hypothesis (Callaghan and Tottenham, 2016), which proposes that EA accelerates the development of the fear circuitry in order to adapt to harsh surroundings and therefore confers an important evolutionary advantage in adverse, stressful environments (Callaghan and Tottenham, 2016). Studies have provided data supporting the Stress Acceleration Hypothesis (Callaghan and Tottenham, 2016) by showing that EA accelerated the development of the frontoamygdala connectivity in children exposed to parental deprivation in such a way that prefrontal cortex-amygdala interactions were more “adult-like” following EA when compared to children not exposed to parental deprivation (for a review, see Gee et al., 2013). When studied in a laboratory context, such differences in cognitive processes may be perceived as abnormal. However, new data by Ellis et al. (2017) suggest that these cognitive changes could be evolutionary adaptive so that EA may shape social and cognitive abilities in order to better adapt to a threatening environment. For example, if a child is reared in an early violent environment, it may be evolutionary adaptive to take a longer time to regulate a negative affect or to present an attentional bias toward threat, as it may allows quicker reaction to threat (Shackman et al., 2007). However, such cognitive adaptation could lead to the development, maintenance and/or exacerbation of vulnerability to stress and anxiety in adulthood (for a review, see Raymond et al., 2018).

The current study, combined with a previous psychoneuroendocrine study conducted on the sample of healthy adults exposed to EA, suggest that minimal age at exposure might influence whether or not the acceleration of the frontoamygdala connectivity occurs. Indeed, in a previous study, we supported the Stress acceleration hypothesis by showing that first exposure to EA during the development of the amygdala (between 3 and 7 years old) led to increased cortisol awakening response and decreased cortisol reactivity to a laboratory psychosocial stressor as opposed to those first exposed between 0 and 2 or after 8 years old (Raymond et al., 2021). This study therefore suggests that first exposure to EA *during* the development of the amygdala but *before* the frontoamygdala connectivity is developing adaptively alters the activity of the hypothalamic-pituitary-adrenal axis (Raymond et al., 2021). In the current study, we suggest that being exposed to EA for the first time *after* 8 years of age (during development of frontoamygdala connectivity) may prevent the acceleration of the frontoamygdala connectivity, resulting in decreased prefrontal cortex inhibition of the amygdala during threat perception.

From a clinical perspective, it is also possible to believe that first exposure to EA during late childhood/adolescence leads to long-lasting (clinical or subclinical) cognitive vulnerability to anxiety in women. Indeed, various studies have suggested that adolescence represents a vulnerable window in the development of various anxiety disorders accompanied by an increased attentional bias toward threat (Derryberry and Reed, 2002) such as social phobia, panic disorder, agoraphobia and generalized anxiety disorder, all of which are twice as prevalent in women as opposed to men (Beesdo et al., 2009). Although further studies are needed in order to better understand the specific mechanism underlying this increased increase incidence of anxiety disorders in adolescence, studies have suggested that an interaction between individual factors (genetic, neurobiological, and temperament) combined with chronic stress (such as EA) could be at play (for a review, see Beesdo et al., 2009). Here, we suggest that healthy women who did not (or not yet) develop anxiety disorders following exposition to EA during adolescence might still present cognitive vulnerabilities that are typically found in children, teenagers and adults who suffer from such psychopathologies. It would be interesting for future studies to investigate the psychosocial, endocrine and neurocognitive protective factors that might prevent the development of psychopathologies in adolescence following exposure to EA. By doing so, interventions could focus on promoting these protective factors following exposition to EA in youth.

We also report that groups did not differ in implicit emotionregulation as measured by the *Emotional conflict task* (Etkin et al., 2006), a task believed to be sustained by the prefrontal cortex and frontoamygdala connectivity. Theoretically, if exposure to EA after the age of 8 leads to poorer frontoamygdala connectivity as proposed above, then adults from the group exposed to EA after the age of 8 should have presented an impaired performance on this task. However, it has to be noted that very few studies have provided cognitive data on the *Emotional conflict task* in populations exposed to EA, and it is possible that this task does not tap entirely on cognitive processes sustained by prefrontal cortex. To this day, only one study has found that children exposed to trauma performed poorly on the *Emotional conflict task* when compared with matched non-exposed children (Marusak et al., 2015). Second, the complexity of the network implying emotion regulation (Goldin et al., 2008) could explain the absence of a group difference on the *Emotional conflict task*. Indeed, although the prefrontal cortex and the amygdala are the main brain structures involved in emotion regulation, a wide array of other brain regions are also important in order to allow such regulation to occur (Goldin et al., 2008). One of these brain regions is the hippocampus (Zhu et al., 2019), which begins to develop at birth (Lupien et al., 2009), allowing the contextualization of emotional cues in order to regulate the affect (Goldin et al., 2008). It is therefore possible that being exposed to EA during early development may also affect the ability to regulate emotions in adulthood. In support of this idea, we found that men exposed to EA between 0 and 2 years-old (“Infancy”: development of the hippocampus), presented decreased scores on the “Adjusting” subscale of the *Affective Style Questionnaire* (Hofmann and Kashdan, 2010), which suggests a reduced tendency to reappraise negative emotions.

Our study contains a number of limitations that need to be addressed. First, although we found promising results in terms of the cognitive processes affected by minimal age at exposure to EA, the absence of neuroimaging measures does not allow us to discuss the mechanism(s) underlying our findings. It would be interesting to conduct neuroimaging studies in order to compare frontoamygdala connectivity of adults who were first exposed to EA before and after the age of 8. This would help in further supporting the interpretation of our findings. Second, although self-reported measures of EA have been demonstrated to be valid and reliable [for a review, see Hardt and Rutter (2004)], it is possible to believe that our measure of minimal age at exposure might have been biased by a poor memory recollection of the events. That could be especially the case when assessing events that occurred during the first years of life. Third, although our limited sample size did not allow to address this specific question, it would also be interesting for future studies to investigate whether the nature of EA impact the obtained results. Fourth, the absence of a control group (with a summed score of 0 on the ACE-Q) limits our interpretation of the results. Finally, our sample was predominantly composed of women and sex was not distributed equally across groups. It is possible that the sex differences we observed results from this imbalance in groups composition as opposed to a differential effect of minimal age at exposure to EA in men and women.

To conclude, our results partly support the Life Cycle Model of Stress and suggest that exposure to EA after 8 years old leads to attentional biases in adult women. Drawing on the core assumptions of the Stress acceleration hypothesis (Callaghan and Tottenham, 2016), we argued that such differences could be due to the chronic secretion of stress hormones following first exposure to EA during the development of the frontoamygdala connectivity. This finding is of high importance, given that adolescence was repeatedly shown to be a critical window in the development of anxiety disorders in women. Here, we suggest that exposition to EA during this critical period might lead to a long-lasting cognitive vulnerability to anxiety in healthy women. While this suggests that EA might be an important environmental factor in the development of cognitive patterns associated with anxiety in women, it also raises the need to further investigate the protective factors that made these individuals resilient toward the disease. By doing so, we could optimize treatment and, ultimately, implement preventive measures for this vulnerable population.

## Data Availability Statement

The raw data supporting the conclusions of this article will be made available by the authors, without undue reservation.

## Ethics Statement

The studies involving human participants were reviewed and approved by the Ethics Committee of the Research Center of the Institut Universitaire en Santé Mentale de Montréal. The patients/participants provided their written informed consent to participate in this study.

## Author Contributions

CR and SL designed the study protocol. CR, VW, A-AJ, and CL collected the data. CR, SL, and M-FM analyzed the data and wrote the manuscript. All authors contributed to the article and approved the submitted version.

## Conflict of Interest

The authors declare that the research was conducted in the absence of any commercial or financial relationships that could be construed as a potential conflict of interest.
